# Extraction of a Weak Flow Field for a Multi-Rotor Unmanned Aerial Vehicle (UAV) Using High-Speed Background-Oriented Schlieren (BOS) Technology

**DOI:** 10.3390/s22010043

**Published:** 2021-12-22

**Authors:** Xianglei Liu, Tongxin Guo, Pengfei Zhang, Zhenkai Jia, Xiaohua Tong

**Affiliations:** 1 Key Laboratory for Urban Geomatics of National Administration of Surveying, Mapping and Geoinformation, Beijing University of Civil Engineering and Architecture, Beijing 100044, China; 2108160219001@stu.bucea.edu.cn (T.G.); 2108570020105@stu.bucea.edu.cn (P.Z.); 2108160220007@stu.bucea.edu.cn (Z.J.); 2 Shanghai Key Laboratory for Planetary Mapping and Remote Sensing for Deep Space Exploration, Tongji University, Shanghai 200092, China; xhtong@tongji.edu.cn

**Keywords:** flow field, background-oriented schlieren, particle image velocimetry, UAV, weak airflow structures

## Abstract

To optically capture and analyze the structure and changes of the flow field of a weak airflow object with high accuracy, this study proposes novel weak flow field extraction methods based on background-oriented schlieren. First, a fine background pattern texture and a sensor network layout were designed to satisfy the requirement of weak flow field extraction. Second, the image displacement was extracted by calculating the correlation matrix in the frequency domain for a particle image velocimetry algorithm, and further calculations were performed for the density field using Poisson’s equation. Finally, the time series baseline stacking method was proposed to obtain the flow field changes of weak airflow structures. A combustion experiment was conducted to validate the feasibility and accuracy of the proposed method. The results of a quad-rotor unmanned aerial vehicle experiment showed that the clear, uneven, and continuous quantitative laminar flow field could be obtained directly, which overcame the interference of the weak airflow, large field of view, and asymmetrical steady flow.

## 1. Introduction

At present, the unmanned aerial vehicle (UAV) industry has a wide range of application prospects in both military and civilian operations [1]. With the effectiveness advantages such as taking off and landing without a runway, autonomous operation, and a wide scope of work, multi-rotor UAVs, as the most commonly used type of UAV, are widely employed in military reconnaissance [2,3,4], military strikes [5], disaster rescue, tilt photography imaging, agricultural applications [6,7], logistics transportation, exploration, and other fields [8,9]. However, due to the many rotating lift surfaces [10], strong coupling interference [11], obvious unsteady flow, and poor stability [12] of multi-rotor UAVs, it is essential to understand the mechanisms of their aerodynamic characteristics. Although many engineering prototypes and products exist for current multi-rotor UAVs, the mechanism research is still at the stage of simulation experiments, and it is necessary to obtain real flow field data to further expand the research [13,14].

In general, the structural change characteristics of a flow field are important parameters for UAV stability analysis and structural optimization design. Therefore, special types of equipment and technology have been used to study the characteristics of UAV flow fields, including flow sensors and flow visualization methods. Because it can directly obtain a continuous flow field and a detailed vortex structure, flow visualization technology has become the main research method for studying a flow field. Moreover, in addition to the visual expression of fluid changes, qualitative and quantitative analyses have further gradually emerged to achieve the acquisition and analysis of flow phenomena. In general, flow visualization technologies are mainly divided into three categories [15]: (1) Adding tracer particles: Some particles that are easy to track and locate in the area of influence of the experimental fluid are added, and the movement state of the fluid is displayed by these tracer particles [16]. (2) Surface adhesion coating: Some coating materials (such as pressure-sensitive coating) are attached to the surface of the object to be tested in a wind tunnel experiment. The surrounding flow will change the color of the coating, thereby enabling the analysis of the flow characteristics of the fluid [17]. (3) Optical-based flow visualization method: Compared with the above two types of flow visualization methods, the optical-based flow visualization method uses the deflection characteristics of light in a flow field to display the flow of a fluid and the physical parameters of a flow field through optical changes [18,19,20]. The optical-based flow visualization method has many advantages: (1) Non-contact measurement does not require the addition of tracer particles or additional sensors in the flow field; so, the flow field changes are not affected; (2) Quantitative analysis, which can not only provide an intuitive and vivid flow map but also allow some quantitative measurement data to be obtained for the flow field; (3) with the upgrading of optical equipment such as the improvement of the resolution of cameras, it has become easier to obtain high-precision measurement results [21].

In general, optical-based flow field visualization methods include traditional schlieren technology and background-oriented schlieren (BOS) technology [22]. The traditional schlieren method uses the light deflected by the knife edge cutting diaphragm so that the change of the projected light intensity can be observed in the field of view to achieve the visualization of the flow field, but there are significant limitations on the actual field of view size. Moreover, special customized light paths and equipment are also required, which are expensive [23]. BOS technology is an emerging flow visualization method that can be used to obtain the first derivative of density based on the light deflection and flow field density changes caused by flow field disturbances, in combination with high-speed video measurement methods [24]. It is important to obtain the first derivative of density to perform a quantitative analysis of the flow field changes and correction of aero-optical effects [25]. Moreover, for the density gradient through a given deflection field, the boundary conditions can be solved with Poisson’s equation obtained with the finite element method. Therefore, the quantitative refractive index distribution can be obtained, and the quantitative density field distribution can be obtained using the Gladstone–Dale equation. Over the past 10 years, BOS technology has been frequently used for flow field qualitative analysis and quantitative measurement [26] and has thus become an important basic method for flow field measurement.

The core of BOS technology is to accurately obtain the relationship between the background deflection and the first derivative of the density. Therefore, it is important to obtain the background deflection, which should affect the accuracy of the flow field. At present, particle image velocimetry (PIV) and optical flow are the two main methods used to determine BOS deflections [27,28,29]. Two mainstream optical flow algorithms are Lucas–Kanade (L–K) optical flow and Farneback optical flow methods [30,31]. The L–K optical flow method calculates optical flow based on feature points and two basic assumptions, and its calculation results are accurate, simple and efficient. However, due to the sparse and uneven distribution of feature points, only sparse optical flow field changes can be obtained. The Farneback optical flow method flow uses quadratic polynomial to approximate each neighborhood of frames to obtain the field changes of dense optical flow. However, the calculation is complex and time-consuming, and the global calculation also introduces errors, resulting in the decline of accuracy. PIV is an efficient and convenient particle image processing method that can quickly extract the displacement and velocity fields of particles. In principle, high particle density is required for the PIV algorithm, which is difficult to accomplish in complex or high-strength flow fields [32]. However, there are high-density artificial speckles for the BOS method that satisfy the above conditions. The effect of the cross-correlation algorithm calculated with the PIV is relatively good, and the method is used to calculate the offset in the experiment.

However, there are two limitations for the current BOS methods. One limitation is that the resolution of the BOS system is easy to be affected by the experimental parameters, and the detection objects need to cause large flow field structure disturbance (super wind tunnels and flame or high-temperature objects in the laboratory). These objects are relatively easy to detect and less affected by the experimental parameters, as the characteristics of flow field structure are obvious or basically symmetrical. However, for the weak flow field, there is no appropriate standard to determine the experimental parameters, including the parameters of the background plate and the parameters of the sensor arrangement, so it is difficult to arrange an appropriate experimental device for the experiment. The other limitation is that high frame rate BOS data have a high spatio-temporal resolution, but there are few methods to conduct spatio-temporal analysis and verification of the results, including visual interpretation directly according to the displacement change for the comparison of multi-frame analysis results [33,34] and single baseline numerical analysis in the area with severe change or obvious characteristics [35]. As the structural changes of UAV weak flow field are not obvious between the adjacent frames, it is difficult to make spatio-temporal analysis and verification using visual interpretation and simple baseline analysis methods. Therefore, to extract the flow field structure of the weak wind object accurately, a fine background pattern texture and sensor network layout are presented to build a BOS system construction standard suitable for weak flow field in this study. Moreover, a time series baseline accumulation verification analysis method is proposed to make spatio-temporal analysis and verification for weak flow field.

## 2. BOS Technology Measurement Principle

The air medium should satisfy the Gladstone–Dale equation between the refractive index and the density of the flow field. Therefore, the density of the flow field can be obtained by calculating the refractive index of the flow field, as follows:

(1) n−1=Kρ

where n is the refractive index, K is the Gladstone–Dale constant, and ρ is the density of the flow field.

**Figure 1:**
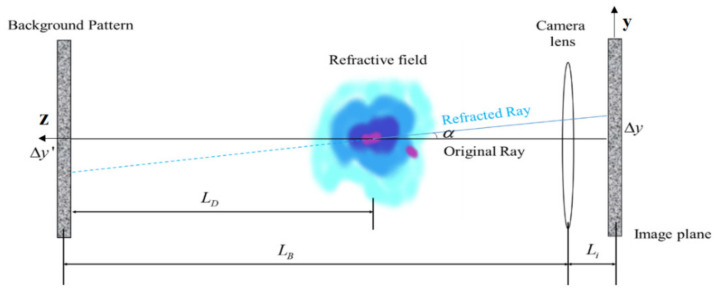
Simplified model of light deflection in the refractive index field in a background-oriented schlieren setting. Black solid line: optical path without refractive index field. Blue solid line: light path with refraction field. The blue dotted line represents the virtual path of the light.

Figure 1 shows a schematic view of the optical path for BOS measurement, which contains two steps. First, a reference image is generated by recording the background pattern without the undisturbed flow field. As shown in Figure 1, the black solid line is an optical path without refraction, which is the projection of the background pattern without the disturbance of the flow field. Then, the background pattern affected by the density of the flow field is photographed as an analysis image. As shown in Figure 1, the blue solid line is an optical path with the refraction field, which is the deflection of the light caused by the disturbance of the flow field. Finally, the image difference of background pattern can be determined between the reference image and the analysis image using the PIV or OF methods, and the displacement Δy of the background pattern can be obtained with the deflection angle $\alpha_{y}$ in the direction y, which is caused by light deflection, as follows:

(2) $$\Delta y=L_{D}\frac{L_{i}}{L_{B}}\alpha_{y}$$

where $L_{D}$ is the distance between the background plate and the refractive field, $L_{B}$ is the distance between the background plate and the camera lens, and $L_{i}$ is the distance between the camera lens and the image plane.

The light passes through the flow field along the Z axis. dz is the differential along the Z axis. The deflection angle α is projected on the *X*-*Z* coordinate plane to obtain the deflection angle of the light in the y direction. For the air medium, the change of the refractive index caused by the flow field disturbance can be ignored. When calculating the deflection angle $\alpha_{y}$, the refractive index can be regarded as an environmental constant $n_{0}$.

(3) $$\alpha_{y}=\frac{1}{n_{0}}\int \frac{\partial n}{\partial y}dz$$

The imaging system has to be focused on the background pattern, which satisfies the following formula:

(4) $$\frac{1}{f}=\frac{1}{L_{i}}+\frac{1}{L_{B}}\Rightarrow L_{i}=\frac{L_{B}f}{L_{B}-f}$$

where f is the focal length, $L_{i}$ can be represented with $L_{B}$ and f, and the displacement Δy of the background pattern can be rewritten as follows:

(5) $$\Delta y=L_{D}\frac{f}{L_{B}-f}\alpha_{y}$$

By combining Equations (3) and (5), the relationship between the displacement Δy of the background pattern and the refractive index n of the flow field can be obtained:

(6) $$\Delta y=(\frac{L_{D}}{L_{B}-f})\frac{f}{n_{0}}\int \frac{\partial n}{\partial y}dz$$

According to Equation (6), the larger the $L_{D}$, the greater the displacement of the background pattern and the higher the sensitivity of the system, which makes it easier to detect the low-density gradients of the flow field. However, with the increase of the system sensitivity, the resolution of the background pattern decreases, which reduces the calculation accuracy of the displacement in the search window area with the cross-correlation algorithm. Therefore, it is necessary to ensure a suitable experimental distance according to the flow field and the actual layout of the measurement object.

The relationship between the displacement Δy of the background pattern and the density gradient can be obtained by Equation (1), as follows:

(7) $$\Delta y=(\frac{L_{D}K}{L_{B}-f})\frac{f}{n_{0}}\int \frac{\partial \rho }{\partial y}dz$$

When the density field is two-dimensional or approximately two-dimensional, it can be considered that the density gradient remains unchanged along the optical axis. When the width of the disturbed flow field is L, the relationship between the density gradient and the displacement Δy of the background pattern can be obtained as follows:

(8) $$\frac{\partial \rho }{\partial y}=L(\frac{L_{B}-f}{L_{D}K})\frac{n_{0}}{f}\Delta y$$

Similarly, the relationship between the displacement and the density of the background pattern in the X direction can be obtained as follows:

(9) $$\frac{\partial \rho }{\partial x}=L(\frac{L_{B}-f}{L_{D}K})\frac{n_{0}}{f}\Delta x$$

Consequently, the density gradient of the flow field satisfies the quantitative relationship shown in Equations (7) and (8); so, the density gradient of the flow field can be calculated by obtaining the displacement of the background pattern in the experimental images.

## 3. Methodology

**Figure 2:**
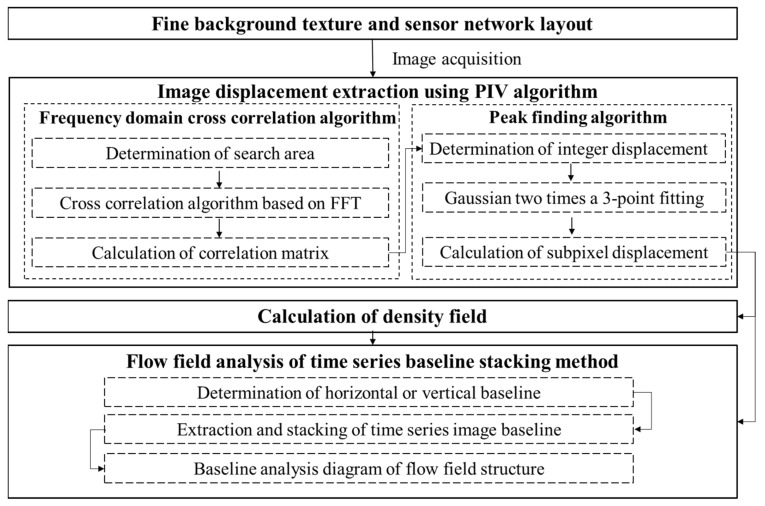
Framework of the entire methodology.

The overall concept of the proposed method for obtaining the mechanism of the weak airflow field is shown in Figure 2. The concept can be summarized with the following four aspects: (1) Fine background pattern texture and sensor network layout, (2) Image displacement extraction using the PIV algorithm, (3) Calculation of the density field, and (4) flow field analysis using the time series baseline stacking method.

### 3.1. Fine Background Texture and Sensor Network Layout

To extract the flow field structure of the weak wind object accurately, an appropriate background plate design (including background contrast, number of points, and point size) and the layout of the background plate and camera were two important factors. At present, different experiments have their own appropriate experimental background pattern and layout setups [36]. However, these setups should satisfy the following basic requirements: (1) The background pattern should have a clear contrast, such as black dots on a white background or white dots on a black background. (2) The number of generated background points should be sufficient to ensure accuracy. (3) A sufficient distance should be kept between the experimental background and the flow field. Therefore, it may be more effective to set a high-density speckle background for the flow field monitoring of weak airflow and further validate the suitable instrument layout.

**Figure 3:**
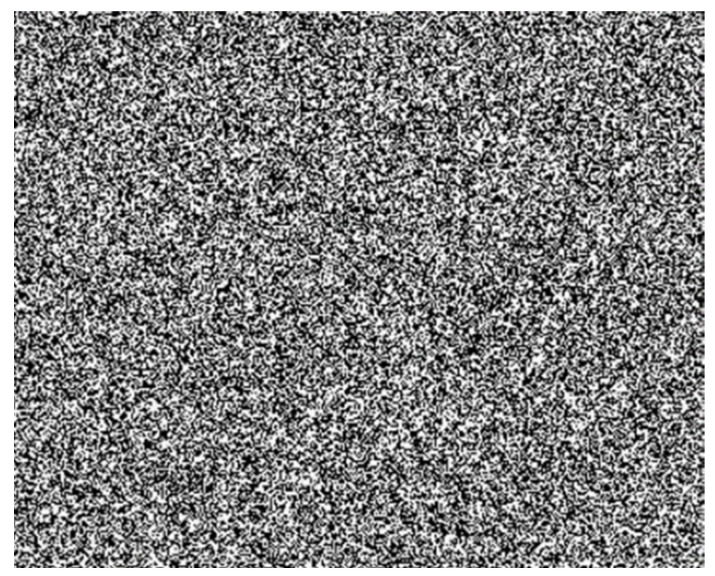
Designed speckle background.

According to Equation (4), the resolution of a BOS system is affected by the distance $L_{D}$ between the background plate and the disturbed flow field. Increasing $L_{D}$ will increase the light deflection, which should make it easier to detect the low-density gradient of the flow field. Therefore, in this study, to extract the flow field structure of the weak wind object, the speckle background was designed, as shown in Figure 3. The parameter requirements of the background pattern and the experimental layout distance are presented in Table 1.

### 3.2. Image Deflection Extraction Using PIV Algorithm

#### 3.2.1. Cross-Correlation Algorithm

**Figure 4:**
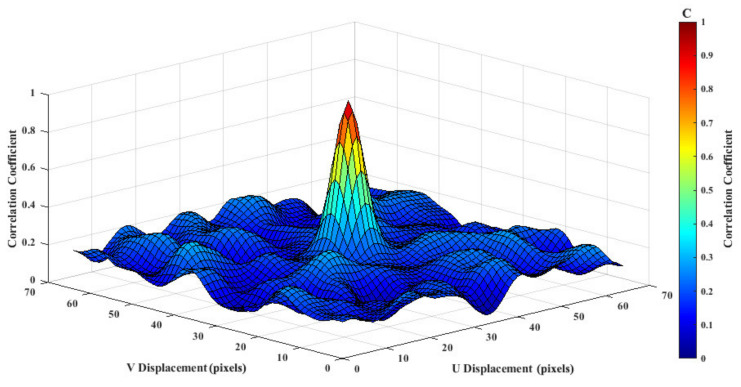
Mean normalized correlation function (sample image of size 65 × 65 pixels).

The cross-correlation algorithm is the key technique for background deflection calculation using PIV. Each image is divided into partially overlapping small sub-images (search area). The search area selected from the reference image is represented as $W_{1}$, and the search area selected from the analysis image is represented as $W_{2}$. Essentially, the cross-correlation algorithm uses a statistical image matching method to find the background pattern deflection from the reference image search area $W_{1}$ to the analysis image search area $W_{2}$. By calculating the correlation function C(m,n) between each image sequence and the search window of the reference image, the position of the peak of the correlation coefficient represents the displacement of the background pattern. The correlation function C(m,n) is obtained as follows:

(10) $$C(m,n)=\sum_{i}\sum_{j}W_{1}(i,j)W_{2}(i-m,j-n)$$

where *i* and *j* denote the image plane coordinates in the Χ and Y directions. Equation (9) is too sensitive to the change of the search window in practical applications, and it can be easily affected by the background noise. Therefore, in this study, the mean normalized cross-correlation was adopted to highlight the peak and reduce noise interference.

(11) $$C(m,n)=\frac{\sum_{i}\sum_{j}\left[W_{1}(i,j)-\bar{W_{1}}][W_{2}(i-m,j-n)-\bar{W_{2}}\right]}{\sqrt{\sum_{i}\sum_{j}{\left[W_{1}(i,j)-\bar{W_{1}}\right]}^{2}\sum_{i}\sum_{j}{\left[W_{2}(i-m,j-n)-\bar{W_{2}}\right]}^{2}}}$$

where $\bar{W_{1}}$ and $\bar{W_{2}}$ are the mean values of $W_{1}$ and $W_{2}$, respectively. Figure 4 shows the correlation plane obtained with the mean normalized cross-correlation algorithm. The peak position is the position with the highest correlation, and it represents the most likely background pattern displacement from the reference image $W_{1}$ to the analysis image $W_{2}$.

In contrast to correlation matrix calculation directly in the spatial domain, PIV is used to calculate the correlation matrix in the frequency domain using a Fast Fourier Transform, which can greatly improve the search efficiency. The correlation theorem states that the cross-correlation of two functions is equivalent to a complex conjugate multiplication of their Fourier transforms:

(12) $$C(m,n)\Leftrightarrow FFT^{-1}({\hat{W}}_{1}\cdot {\hat{W}}_{2}^{*})$$

where ${\hat{W}}_{1}$ and ${\hat{W}}_{2}$ are the Fourier transforms of the functions $W_{1}$ and $W_{2}$, respectively, ${\hat{W}}_{2}^{*}$ is the conjugate complex root of ${\hat{W}}_{2}$, and $FFT^{-1}$ is the inverse Fourier transform.

#### 3.2.2. Peak Finding Algorithm

**Figure 5:**
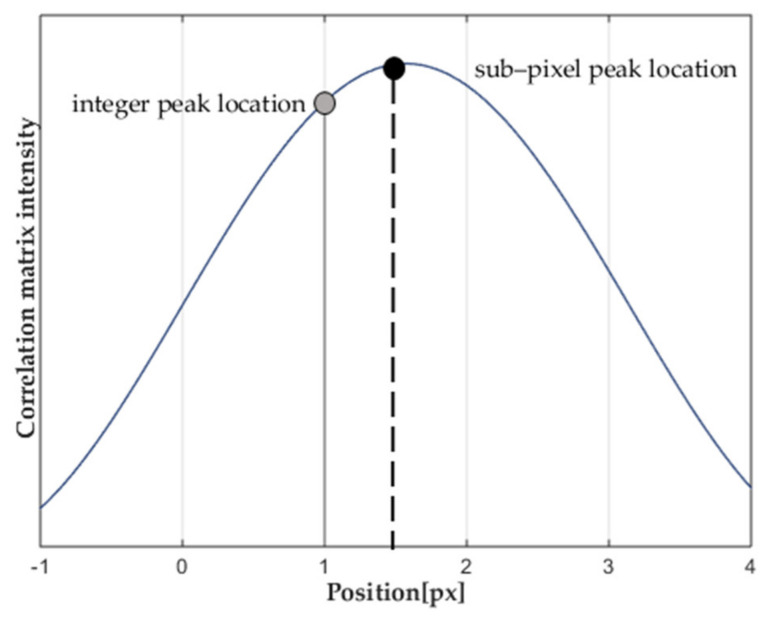
Principle of the Gaussian 2·3-point fit.

It requires not only the correlation algorithm but also the sub-pixel peak finding algorithm to obtain the fine displacement of the background pattern. Therefore, the peak finding technology is used to ensure the sub-pixel precision of image deflections. The background images’ intensity profile formed by the particles on the imaging array can be best described by a Gaussian shape [32]. BOS background speckle is similar to the particle imaging model, so the shape of its correlation peak is also a Gaussian function. The integer displacement can be determined directly between the two search windows based on the location of the peak position, and further calculate the non-integer intensity distribution by a twice three-point fit (Equation (13)) to refine the location of peak with sub-pixel precision. The twice three-point method performs one-dimensional “fitting” through the highest value and its two neighborhoods (three-point estimator), and measures the *X*-axis and *Y*-axis, respectively, for twice fitting, which can better conform to the peak function, as shown in Figure 5.

It is assumed that an integer peak was found at $\left(x,y\right)$ in a correlation function C(m,n) and that the correlation peak satisfies the Gaussian distribution. Then, the accurate location of the correlation peak ${(x}_{sub}{,y}_{sub})$ can be obtained as follows:

(13) $$\begin{array}{l} x_{sub}=x+\{\log C(x-1,y)-\log C(x+1,y)\}\times {\left\{2[\log C(x-1,y)+\log C(x+1,y)-2\log C(x,y)]\right\}}^{-1}\\ y_{sub}=y+\{\log C(x,y-1)-\log C(x,y+1)\}\times {\left\{2[\log C(x,y-1)+\log C(x,y+1)]-2\log C(x,y)\right\}}^{-1} \end{array}$$

After obtaining the accurate location of the correlation peak ${(x}_{sub}{,y}_{sub})$, the displacement ΔxΔy of the background pattern can be obtained by subtracting the center position of the window (half of the window).

### 3.3. Calculation of Density Field

The displacements Δy and Δx of the background pattern are obtained with the PIV algorithm described in the previous section, and the density gradient field of the flow field is obtained by Equations (8) and (9). However, density is an indispensable and important piece of information in practical engineering and experimental research. Therefore, as described in this section, the density field information for the flow field to be measured is calculated using the displacement of the background pattern. If the partial derivatives of the whole displacement vector field are obtained in the X and Y directions, the following Poisson equation can be obtained:

(14) $$\frac{\partial^{2}\rho }{\partial x^{2}}+\frac{\partial^{2}\rho }{\partial y^{2}}=L(\frac{L_{B}-f}{L_{D}K})\frac{n_{0}}{f}(\frac{\partial \Delta x}{\partial x}+\frac{\partial \Delta y}{\partial y})$$

The constant on the right side of the equation is related to the parameters of the experimental system instrument, and it can be replaced by P, and the density gradient can be replaced by ∇ρ; hence, the equation can be expressed as follows:

(15) $$\nabla^{2}\rho =P\cdot (\frac{\partial \Delta x}{\partial x}+\frac{\partial \Delta y}{\partial y})$$

For the calculated displacement vector field and boundary conditions, the above formula can be solved with the finite difference method or the finite element method to obtain the quantitative refractive index field distribution of the projection integral effect in the measurement area. The quantitative density field information can be calculated with the Gladstone–Dale formula.

### 3.4. Flow Field Analysis Using Time Series Baseline Stacking Method

**Figure 6:**
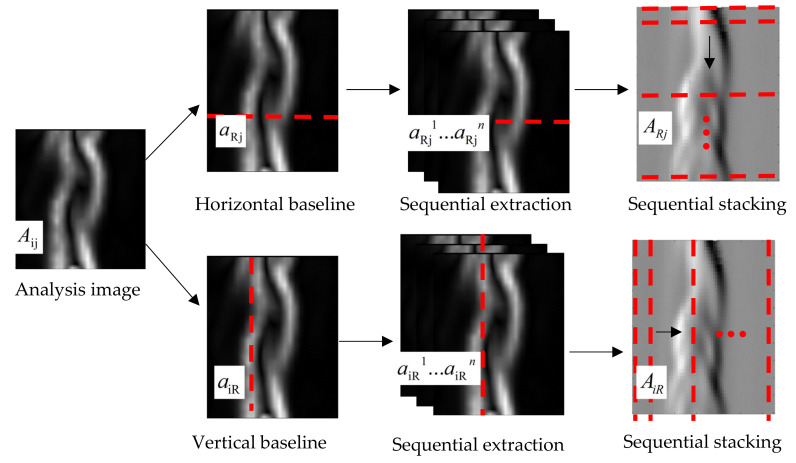
Time series baseline stacking method.

For high-speed BOS, although it has the characteristics of high spatial and temporal resolution, the information for the spatial and temporal changes of the flow field structure in the image is separated. Therefore, it is difficult to display and extract the spatial changes of the continuous time series of flow field structure intuitively. In general, by setting the analysis baselines for the regions of interest and the regions with strong flow field changes, the characteristics of the displacement field changes along the baselines can be obtained, and the temporal and spatial changes of the flow field structure can be displayed with the time series broken line graph of the baseline position changes. However, this kind of analysis requires a large inter-frame difference to create an obvious effect. Because the change of the weak airflow is not obvious, it is difficult to obtain the significant time series change characteristics between two adjacent frames. Therefore, in this study, the baseline stacking analysis method is adopted to obtain the change of the weak airflow, and multiple background schlieren images of the continuous time series are selected for baseline extraction. The extracted baseline matrix is superimposed to form a new time series baseline stacking matrix, which contains the change information for the selected baseline in n consecutive frames. The time series variation characteristics of the flow field structure can be displayed, and the high-precision space–time analysis of the flow field structure can be achieved. Figure 6 shows the model construction and analysis process for the time series baseline stacking method.

The pixel matrix of the flow field analysis image is denoted as $A_{\mathrm{ij}}$. The time series baseline is divided into the horizontal baseline $a_{\mathrm{Rj}}$ and the vertical baseline $a_{\mathrm{iR}}$.

(16) $$A_{\mathrm{ij}}=\left[\begin{array}{ccc} a_{11} & \ldots & a_{1j}\\ \ldots & \ldots & \ldots \\ a_{i1} & \ldots & a_{ij} \end{array}\right]$$

(17) $$a_{\mathrm{Rj}}=\left[\begin{array}{ccc} a_{R1} & \ldots & a_{Rj} \end{array}\right]$$

(18) $$a_{\mathrm{iR}}={\left[\begin{array}{ccc} a_{1R} & \ldots & a_{iR} \end{array}\right]}^{T}$$

The horizontal baseline analysis is used to successively extract the horizontal baselines $a_{\mathrm{Rj}}{}^{1}-a_{\mathrm{Rj}}{}^{n}$ of the region of interest in the image sequence matrices $A_{\mathrm{ij}}{}^{1}-A_{\mathrm{ij}}{}^{n}$ with a frame interval of d.

(19) $$\begin{array}{c} \begin{array}{c} a_{\mathrm{Rj}}{}^{1}=\left[\begin{array}{ccc} a_{Rj}{}^{1} & \ldots & a_{Rj}{}^{1} \end{array}\right] \end{array}\\ a_{\mathrm{Rj}}{}^{1+d}=\left[\begin{array}{ccc} a_{Rj}{}^{1+d} & \ldots & a_{Rj}{}^{1+d} \end{array}\right]\\ \ldots \\ a_{\mathrm{Rj}}{}^{n-d}=\left[\begin{array}{ccc} a_{Rj}{}^{n-d} & \ldots & a_{Rj}{}^{n-d} \end{array}\right]\\ a_{\mathrm{Rj}}{}^{n}=\left[\begin{array}{ccc} a_{Rj}{}^{n} & \ldots & a_{Rj}{}^{n} \end{array}\right] \end{array}$$

A new matrix $A_{Rj}$ is formed by stacking $a_{\mathrm{Rj}}{}^{1}-a_{\mathrm{Rj}}{}^{n}$ from top to bottom, which denotes the horizontal baseline stacking graph for n images, and is given by

(20) $$A_{Rj}=\left[\begin{array}{c} a_{Rj}{}^{1}\\ \ldots \\ a_{Rj}{}^{n} \end{array}\right]$$

The vertical baseline analysis is used to successively extract the vertical baseline $a_{iR}{}^{1}-a_{iR}{}^{n}$ of the region of interest in the image sequence matrices $A_{\mathrm{ij}}{}^{1}-A_{\mathrm{ij}}{}^{n}$ with a frame interval of d.

(21) $$\begin{array}{c} \begin{array}{c} a_{iR}{}^{1}={\left[\begin{array}{ccc} a_{1R}{}^{1} & \ldots & a_{iR}{}^{1} \end{array}\right]}^{T} \end{array}\\ a_{iR}{}^{1+d}={\left[\begin{array}{ccc} a_{1R}{}^{1+d} & \ldots & a_{1R}{}^{1+d} \end{array}\right]}^{T}\\ \ldots \\ a_{iR}{}^{n-d}={\left[\begin{array}{ccc} a_{iR}{}^{n-d} & \ldots & a_{iR}{}^{n-d} \end{array}\right]}^{T}\\ a_{iR}{}^{n}={\left[\begin{array}{ccc} a_{1R}{}^{n} & \ldots & a_{iR}{}^{n} \end{array}\right]}^{T} \end{array}$$

A new matrix $A_{iR}$ is formed by stacking $a_{iR}{}^{1}-a_{iR}{}^{n}$ from left to right, which denotes the vertical baseline stacking graph for n images, and is given by

(22) $$A_{iR}=\left[\begin{array}{ccc} a_{iR}{}^{1} & \ldots & a_{iR}{}^{n} \end{array}\right]$$

## 4. Experiment Results and Analysis

In this study, to obtain a weak airflow field with higher accuracy, a high-speed Complementary Metal–Oxide–Semiconductor (CMOS) transistor camera (CP80-4-M-500, Optronis company, Kehl, Germany), along with a Nikon lens with a focal length of 35 mm (Nikon Corporation, Tokyo, Japan), a high-speed data acquisition card, a workstation, and a tripod, was used to capture the image sequence. The key parameters of the CP80-4-M-500 camera are presented in Table 2.

### 4.1. Displacement Extraction Algorithm Verification

**Figure 7:**
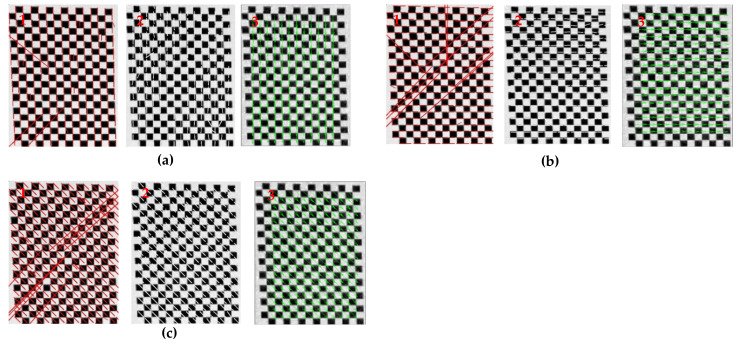
Displacement vector diagrams of checkerboard background. (**a**) Only 2 pixels downward, (**b**) only 2 pixels horizontally to the right, and (**c**) 2 pixels horizontally and vertically together; 1 L–K optical flow, 2 Farneback optical flow, and 3 Cross-correlation.

To verify the accuracy and reliability of the background pattern displacement extraction algorithm, the checkerboard background and artificial speckle background patterns were used to simulate the background displacement. The image size of the checkerboard background was 479 × 624 pixels. To verify the difference between horizontal and vertical directions for the displacement extraction algorithm, three displacement parameters were set for the analyzed image, including moving only 2 pixels vertically downward, moving 2 pixels horizontally to the right, and moving 2 pixels horizontally and vertically together. The commonly used L–K and Farneback optical flow algorithms were compared with the cross-correlation algorithm used in the study. The displacement vector diagrams (expanded by 12 times) are shown in Figure 7, and the evaluation index of displacement extraction is shown in Table 3. The inspection from this figure and table highlights that: (1) The L–K optical flow method produced some larger mismatched results, the resulting root mean squared error (RMSE) was 4.0434 pixels, which greatly reduced the data quality; (2) Farneback optical flow method also produced many wrong displacement value calculations. Its extraction accuracy was only 87.20% for the correct displacement value interval with the threshold of 2 ± 0.2 pixels, and it needed a consumed time of 2.484 s (Table 3). Compared with the above two optical flow methods, the RMSE of the cross-correlation algorithm was less than 0.1 pixel, the extraction accuracy was 100%, and the shortest time was 0.624 s.

**Figure 8:**
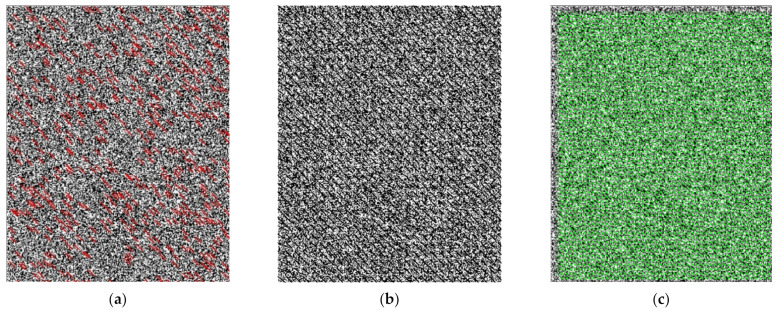
Displacement vector diagrams of the artificial speckle background. (**a**) L–K optical flow, (**b**) Farneback optical flow, and (**c**) cross-correlation.

The image size of the artificial speckle background was 793 × 982 pixels. To ensure the measurement accuracy of micro flow field distribution, the analyzed background image was set to move 1 pixel horizontally and vertically. The displacement vector diagrams are shown in Figure 8 (expanded by 20 times), and the evaluation index of displacement extraction is shown in Table 4. The inspection from this figure and table highlights that: (1) For the L–K optical flow method, there was no large mismatch with an extraction accuracy of 98.37%, and the RMSE was 0.2511 pixels. However, due to the limitations of feature matching, only some uneven sparse optical flows can be obtained in the regions with obvious features. (2) For the Farneback optical flow method, its extraction accuracy was only 90.68% for the correct displacement value interval with the threshold of 1 ± 0.2 pixels, and it needed a consumed time of 4.848 s with the increase of image size and background texture. (3) Compared with the two optical flow methods, the RMSE of cross-correlation algorithm was only 0.1432 pixels, the extraction accuracy was 100%, and the shortest time was 1.024 s. The simulated results of the two kinds of background plates indicated that the cross-correlation algorithm has a good extraction accuracy and time efficiency in displacement extraction for BOS background pattern.

### 4.2. Combustion Experiment

**Figure 9:**
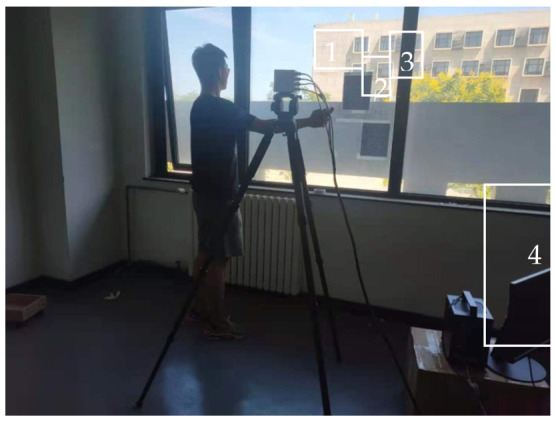
Flame measurement setup. (**1**) High-speed camera with a 35 mm lens. (**2**) Handheld lighter. (**3**) Speckle background plate. (**4**) Data operation and storage industrial control machine (including input and output accessories).

In order to establish a non-uniform refractive index field of the flame, the turbulent flow field above the flame of the lighter was used as the experimental object. According to the requirements of the background schlieren technique and the experimental layout, the distribution density of the experimental background pattern exceeded 60% of the background plate, which could improve the resolution of the weak airflow. The flame density measurement experimental equipment setup is shown in Figure 9. The size of the background plate was 238.5 mm × 195.5 mm, the background plate was directly placed on the transparent glass, and sunlight was used for illumination, which increased the brightness of the background and did not require special light–source illumination. Moreover, the size of the background pattern point in the image had to be greater than 2 pixels. Therefore, the size of the background pattern point setting was about 2–4 pixels in this study. A high-speed CP80-4-M-500 camera was used, along with a Nikon lens with a focal length of 35 mm. The distance between the lens and the background plate was set to about 1 m, and the flame of the lighter was about 0.55 m away from the background, which developed a field of view of 471.0 mm × 351.6 mm. The optical axis of the camera was perpendicular to the background plate, and the camera was focused on the background pattern. The combustion experiment lasted for about 5 s, and the resulting 2500 images were acquired from static to burning for the flame.

To obtain the accurate and dense slow convective flows, the general search area size was 64 × 64 pixels, and the search area size was reduced to 16 × 16 pixels in the weak airflow experiment with a step size of 8 pixels. If the size of the search area was reduced or the step size was increased, the inaccurate results would be sharply increased, and the signal-to-noise ratio would be reduced. Moreover, to avoid analysis algorithm irritations, disturbing elements such as the measurement object and the instruments were masked, for which the values of the flows were equal to zero.

**Figure 10:**
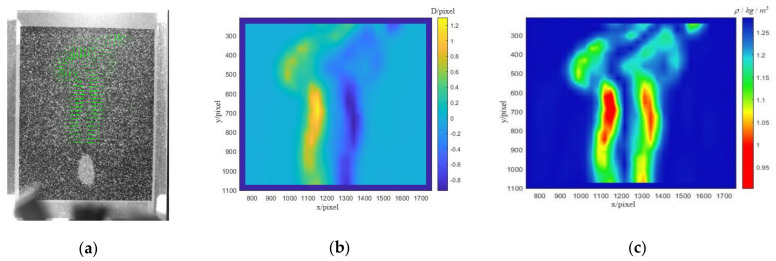
Displacement of flame. (**a**) Background displacement vector diagram, (**b**) horizontal displacement diagram, and (**c**) density field distribution diagram.

With the PIV algorithm described in Section 3.2, a 16 × 16 pixel search area was used to obtain the displacement vector diagram of the background pattern, as shown in Figure 10a. The green arrow in the figure points to the direction of displacement, and the length represents the size of the displacement. The size is displayed in the figure in an eight-fold magnification relationship. The specific numerical relationship was usually split and displayed in the displacement cloud diagram in the horizontal and vertical directions. The main transformation of the flame combustion analysis was the horizontal direction. Therefore, the horizontal displacement that was obtained is shown in Figure 10b. The yellow component in the figure represents the positive displacement to the right, and the blue component in the figure represents the negative displacement to the left. The maximum displacement in the figure is about 1150 pixels in the X direction of positive 1.2 pixels and about 1350 pixels in the X direction of negative 0.8 pixels. Then, the density field was reconstructed, and the density field distribution diagram was obtained, as shown in Figure 10c. It was obvious that the density of the two positions with large displacement transformations changed greatly, which might have been caused by the supplement of the surrounding air after the flame burned oxygen.

**Figure 11:**
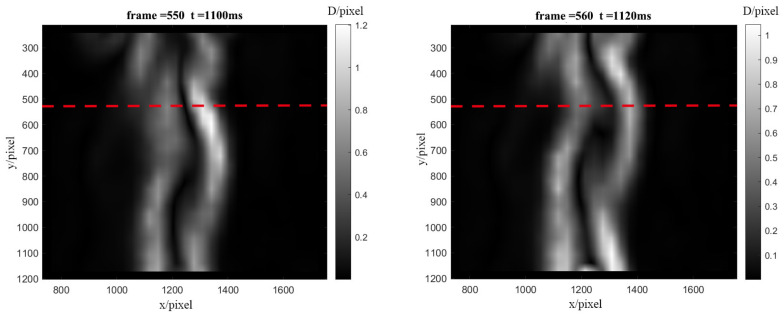

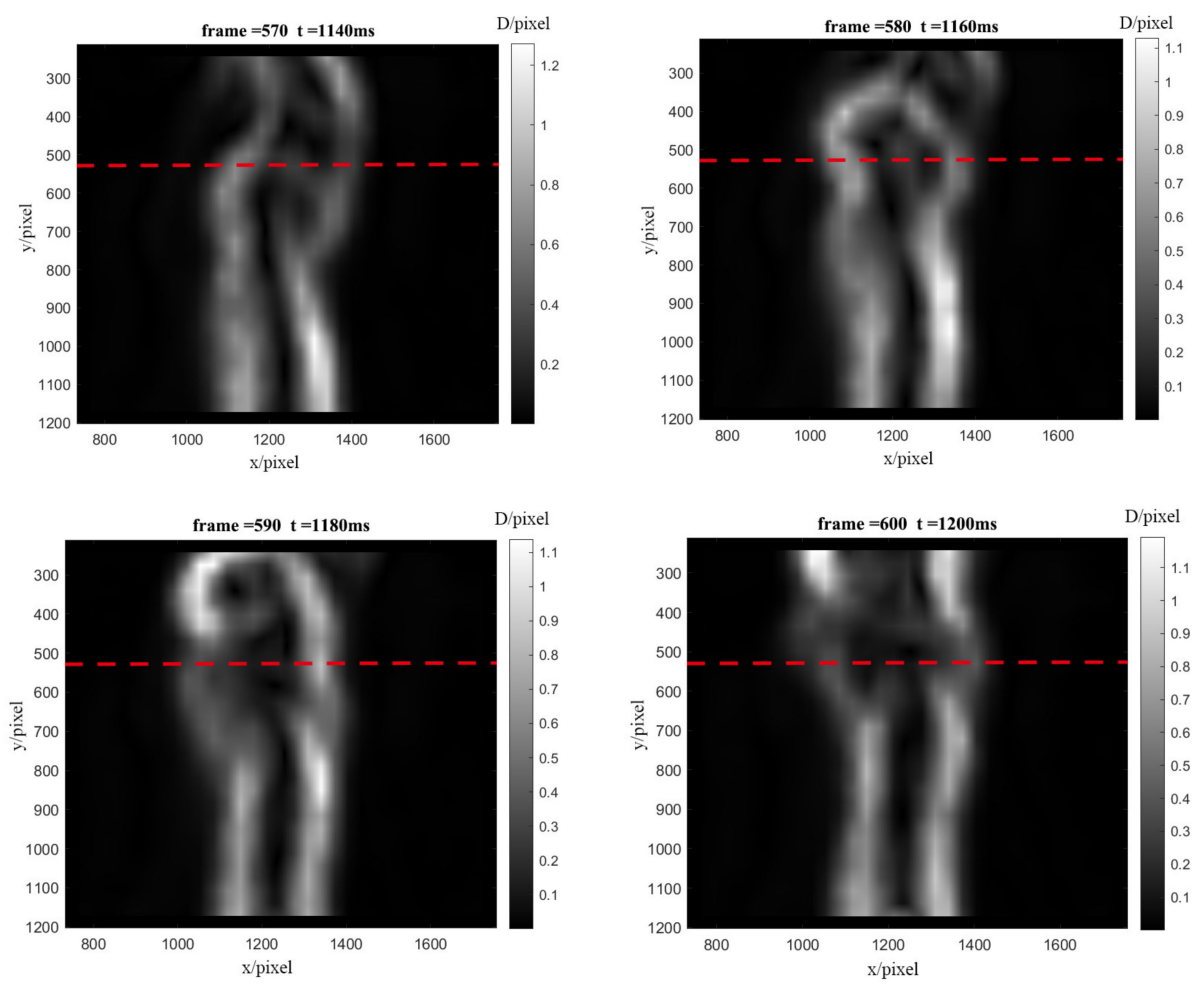
Schematic diagram of frame skipping baseline analysis.

The flow field information for the flame combustion was clearly obtained in the schlieren image, and the flow field changed obviously from ignition to combustion stability. Since the environment was not closed flow, the flame was not a symmetrical structure. The boundary conditions of the flow field above the flame were easy to distinguish, and the structure of the flow field with deflection changed significantly with time and had a certain regularity. However, when the combustion was stable, due to the high shooting frame rate of the high-speed camera, the change of the flow field per frame was small, which was not easy to distinguish. Therefore, in order to extract the temporal change information of the flow field, the analysis method of a frame skipping the extracting baseline and generating a broken line graph was used to extract the maximum value of the baseline position and the overall change trend. As shown in Figure 11, the sequence schlieren images with stable combustion after 550 frames were compared and analyzed at intervals of 10 frames (from frame 550 to frame 600), and the red dotted line was set at 490 pixels (14.3 cm above the flame) in the vertical direction, which was the horizontal analysis baseline.

**Figure 12:**
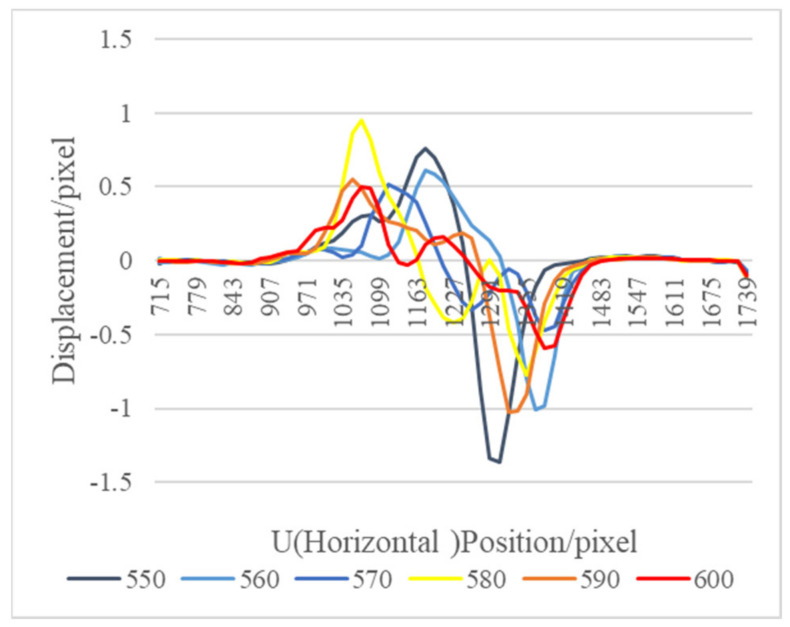
Frame skipping baseline analysis diagram.

The flow field 14.3 cm above the flame was selected as the region of interest for the horizontal analysis. As shown in Figure 12, the peak of the outer flame boundary on the right side of the images from frame 550 to frame 570 of the flow field showed a downward trend, that from frame 580 to frame 590 showed an upward trend, that at frame 600 showed a downward trend again, and the change interval of its flow field was about 10–30 frames. The reason for this change might have been the change of the full combustion position since the indoor airflow caused the combustion air supplement to vary. For a more obvious analysis, the fine structural changes of the flow field above the flame in the continuous frames are shown with the baseline stacking analysis method to obtain the change of the airflow of the flame, which was selected from frames 550 to 600 of the background schlieren images of the continuous time series for baseline extraction.

**Figure 13:**
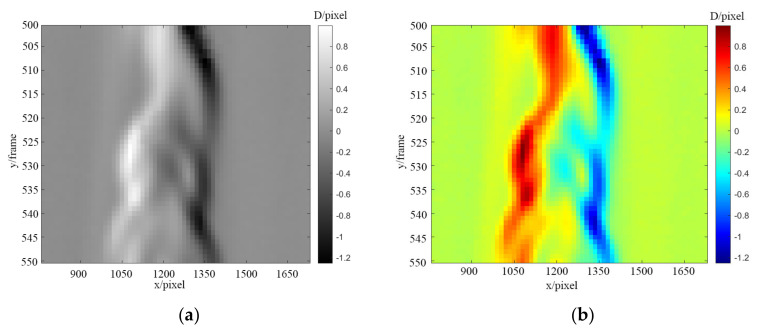
Time series horizontal baseline stacking diagram. (**a**) A grayscale image, and (**b**) a jet color image.

Figure 13 shows the stacking diagram of the horizontal baselines from frames 550 to 600 stacked from top to bottom, and the figure shows the temporal change characteristics of the image and the intensity of the change. The maximum displacement of the flow field was 1.0–1.2 pixels/frame, and the flow field had to be stable in a static state in a symmetrical state. With the action of the indoor circulating flow field, the left and right deviations relative to the center line occurred, which was a normal phenomenon. The method successfully showed the boundary conditions of the flow field, and the intensity change trend was also consistent with the previous analysis of the line graph. The variation law showed that the maximum position of the flow field (baseline analysis position) weakened first, then became stronger, and then became weaker again. The results showed that the time series baseline stacking method could accurately reflect the obvious changes of the weak airflow.

### 4.3. UAV Flow Field Visualization

**Figure 14:**
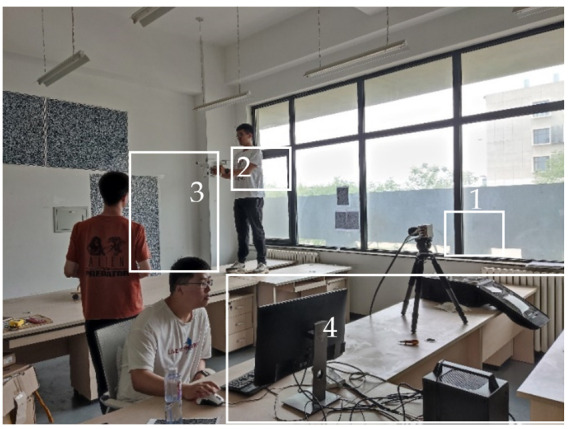
UAV measurement setup. (**1**) High-speed camera and 35 mm lens. (**2**) Handheld UAV. (**3**) Speckle background plate. (**4**) Data operation and storage industrial control machine (including input and output accessories).

In order to establish a non-uniform refractive index field for a quad-rotor UAV, an experimental system was constructed to obtain the flow field structure of a quad-rotor UAV for visualization. As shown in Figure 14, a high-speed camera CP80-4-M-500 was used, along with a Nikon lens with a focal length of 35 mm. The size of the background plate was 1200 mm × 960 mm, and four pieces were used to form a large background plate. The background plate (attached to the wall) was perpendicular to the optical axis of the camera. The distance between the lens of the camera and the background plate was adjusted to 7800 mm, and the distance from the UAV flow field to the background plate was about 4000 mm, which resulted in a field of view of 2516.3 mm × 1878.2 mm. It was almost impossible to achieve that using the traditional schlieren method. The large field of view was more conducive to observing the changes in the UAV’s airflow. In the experiment, two sets of 5 s, 2500-frame UAV flight sequence images were obtained.

**Figure 15:**
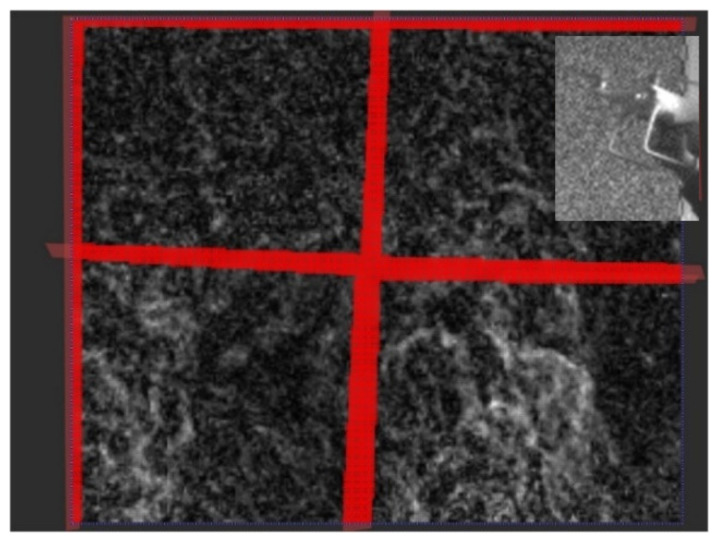
Displacement grayscale diagram of UAV flow field.

In this study, to obtain the displacement of the background pattern with the frequency domain cross-correlation algorithm, the search area was 16 × 16 pixels with a step size of 8 pixels, which could ensure that accurate and dense flow field data were obtained. Since the flow field caused by the four-rotor UAV was complex and difficult to analyze, for the experimental handheld UAV, only the wind flows of the two left rotors were collected and analyzed. The obtained UAV flow field displacement grayscale diagram is shown in Figure 15. Moreover, due to the moving interference of boundary, gap and UAV, it was easy to produce false matching. Therefore, the red area was the masked area to prevent the wrong data from affecting the flow field structure analysis.

**Figure 16:**
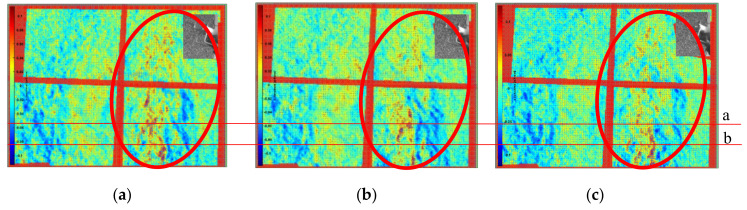
Frame skipping analysis diagram and horizontal baseline setting. (**a**) T = frame 570, (**b**) T = frame 595, (**c**) T = frame 620.

**Figure 17:**
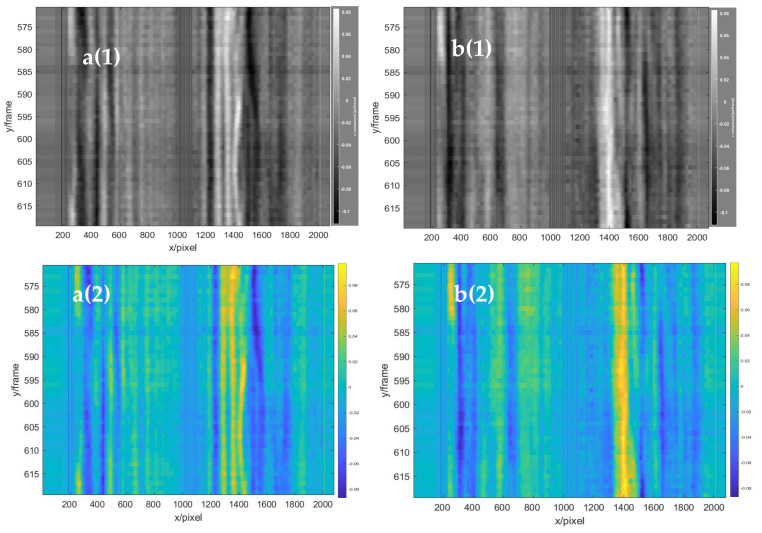
The stacked baseline analysis graphs in the horizontal direction of 50 frames. **a**(**1**) Baseline a (gray scale), and **a**(**2**) baseline a (RGB color). **b**(**1**) Baseline b (gray scale), and **b**(**2**) baseline b (RGB color).

**Figure 18:**
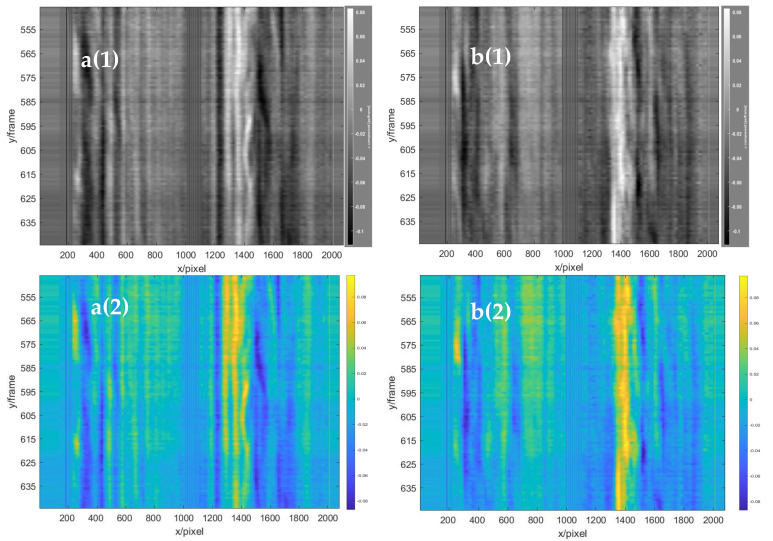
The stacked baseline analysis graphs in the horizontal direction of 100 frames. **a**(**1**) Baseline a (gray scale), and **a**(**2**) baseline a (RGB color). **b**(**1**) Baseline b (gray scale), and **b**(**2**) baseline b (RGB color).

To clearly show the obvious flow field changes, the frame difference of 25 frames was used to obtain the horizontal displacement field of the left double rotor, as shown in the red area in Figure 16. In the figure, the red component on the left in the red circle represents the airflow moving to the right, and the blue component on the right in the red circle represents the airflow moving to the left. Comparison and analysis of the three images revealed that the horizontal flow field of the handheld UAV was relatively stable during the selected time period. Moreover, the fine structural changes of the flow field under the UAV in the continuous frames were shown with the time series baseline stacking analysis method, and frames 570 to 620 of the background schlieren images of the continuous time series were selected for baseline extraction. Two continuous 50-frame images at the two baselines (baselines a and b shown in Figure 16) from frames 570 to 620 were superimposed from top to bottom in the horizontal direction, as shown in Figure 17a(1),b(1). Simultaneously, to facilitate the visual analysis of baseline stacking analysis chart, the analysis chart was stained with imagesc function, as shown in Figure 17a(2),b(2). The maximum displacement of the flow field was 0.1 pixel/frame. The flow field structures of the two baseline superposition diagrams were almost stable within 50 frames, which indicated that the flow field of the horizontal direction under the UAV was relatively stable. Therefore, the horizontal structure of the flow field was continuous and stable at almost the same position. However, there was only a slight deviation, which might have been caused by the vibration of the handheld or indoor disturbed airflow. Moreover, to further evaluate the preciseness of time series baseline stacking method for flow field analysis, the data set was expanded 100 frames, from 545 to 645. As shown in Figure 18, the flow field structure of the two baseline overlay images of 545 to 645 frames was also almost stable, which proved the stable state of the horizontal structure of the UAV flow field.

**Figure 19:**
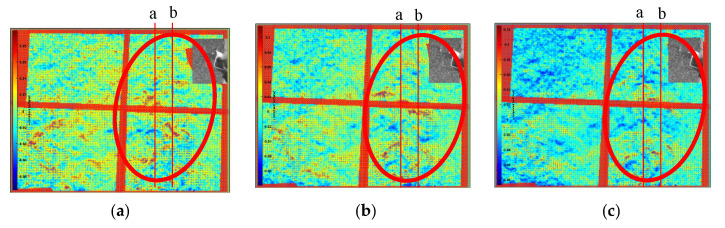
Frame skipping analysis diagram and vertical baseline setting. (**a**) T = frame 570, (**b**) T = frame 595, (**c**) T = frame 620.

**Figure 20:**
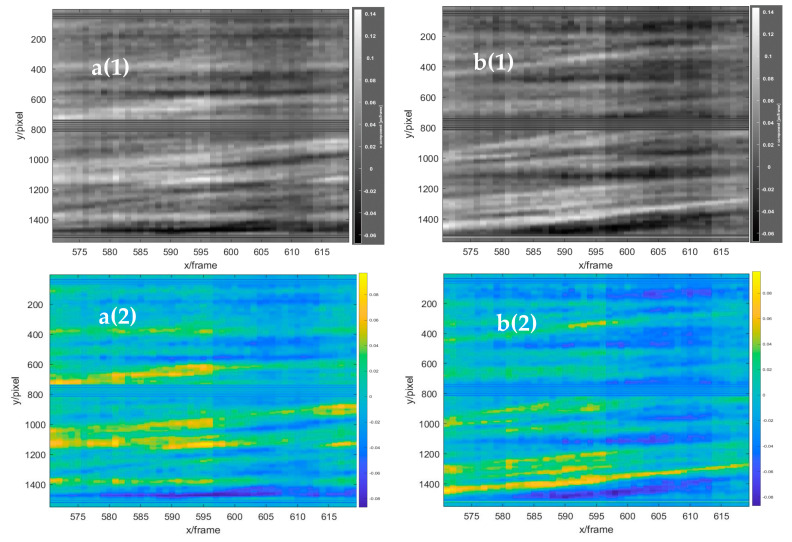
The stacked baseline analysis graphs in the vertical direction of 50 frames. **a**(**1**) Baseline a (gray scale), and **a**(**2**) baseline a (RGB color). **b**(**1**) Baseline b (gray scale), and **b**(**2**) baseline b (RGB color).

**Figure 21:**
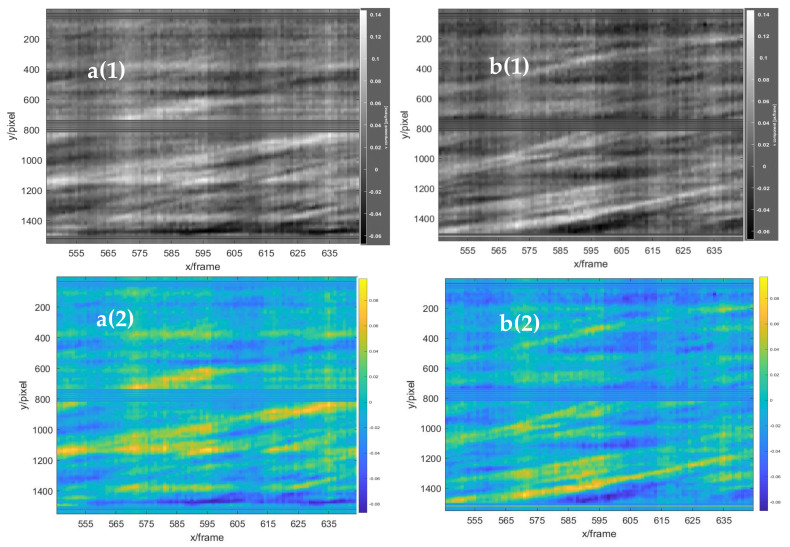
The stacked baseline analysis graphs in the vertical direction of 100 frames. **a**(**1**) Baseline a (gray scale), and **a**(**2**) baseline a (RGB color). **b**(**1**) Baseline b (gray scale), and **b**(**2**) baseline b (RGB color).

The vertical displacement field of the left double-rotor was obtained with the frame difference of 25 frames, as shown in the red area in Figure 19. In the figure, the red component on the left in the red circle represents the airflow moving downwards, and the blue component on the right in the red circle represents the airflow moving upwards. Through the comparative analysis of the three images, it was found that the vertical flow field of the UAV changed significantly, the vertical structure of the flow field changed upward at an interval of 25 frames, and the structure position changed by more than 50 pixels, which represented the vertical lift change of the UAV to maintain its own suspension. Moreover, two continuous 50-frame images at the two baselines (baselines a and b shown in Figure 19) from frames 570 to 620 were superimposed from left to right in the vertical direction, as shown in Figure 20a(1),b(1). Simultaneously, to facilitate the visual analysis of baseline stacking analysis chart, the analysis chart was stained with imagesc function, as shown in Figure 20a(2),b(2). It was found that the characteristics of the strong eddy current generated by the UAV rose continuously with time, which was consistent with the change trend of the 25-frame skipping analysis diagram. The indirect velocity measurement of the BOS flow field could be achieved by using the eddy current positioning analysis method (the displacement was extracted during 50 frames of the strong eddy current position, as the red line shown in Figure 20b(2), which was difficult to achieve with the traditional BOS. The average 50 frames of the strong eddy current structure changed by 205 pixels, and a wind speed of about 4.969 m/s was generated by the two propellers of the UAV. Moreover, to demonstrate the accuracy of the results, the data set was also expanded to 100 frames from 545 to 645. As shown in Figure 21, it changed continuously and maintained a steady rise for the strong eddy current characteristics of the two baseline superimposed images from 545 to 645 frames. The strongly eddy structures changed 288 pixels in 70 frames, as the red line shown in the Figure 21b(2), and the two propellers of the UAV produced wind speeds of about 4.9869 m/s, with only a 17 mm error.

The displacement field generated in the experiment showed that the maximum offset caused by the UAV flow field was about a sub-pixel, and the value of the UAV flow field was small, which made it extremely difficult to capture and display the UAV flow field. The difference between the flow field structure and the environmental disturbance was only one order of magnitude. This made it easy for the flow field to be confused with the environment, and it was also difficult to distinguish the flow field from the environmental noise. However, the structural change law of a strong eddy current was successfully extracted with the time series baseline stacking method, which also fully proved the stability and continuity of the flow field. Therefore, the experimental results showed that the proposed method was effective. The experimental results proved that the weak flow field extraction methods successfully distinguished between environmental disturbance and UAV wind flow, and clear, continuous, and stable results were obtained. 

## 5. Conclusions

Conventional measurement methods are generally constrained by space and equipment for weak wind flow extraction, and strict optical path conditions make it very difficult to perform measurements in outdoor environments. BOS technology is sensitive to the refractive index gradient in a transparent medium, and it can be used for the visualization of objects with weak wind flows. Therefore, in this paper, a novel flow field structure analysis method is proposed for weak airflow. The results presented in the paper clearly highlight the following points.

(1) A fine background pattern texture and a sensor network layout were designed to satisfy the requirement of weak flow field extraction. The results of the combustion and a quad-rotor UAV experiment indicated that the clear, uneven, and continuous quantitative laminar flow field could be obtained directly, which overcame the interference of the weak airflow, large field of view, and asymmetrical steady flow. The results were also validated using the continuity and regularity of the flow field and the consistency of the difference.

(2) By comparing the performance of cross-correlation algorithm with L–K optical flow and Farneback optical flow methods on checkerboard background and artificial speckle background, it was concluded that the cross-correlation algorithm has a good extraction accuracy and time efficiency in displacement extraction for BOS background pattern.

(3) The proposed baseline stacking method was used to obtain the time series change characteristics of the flow field, and the flow field structural change feature was used to inversely calculate the wind speed of the flow field.

(4) With the current goal of weak wind flow experiments in small indoor and outdoor scenarios, the flow field obtained with the weak flow field extraction method could meet the visualization requirements, the sequence data could be stacked and demonstrated by using the relevant baseline, and the temporal and spatial information for the flow field could be displayed with intuitive correlation.

## Figures and Tables

**Table 1:** Parameters of the requirements of the speckle background and the experimental layout.

Parameters	Number of Background Points	Size of Background Points	Layout Distance
	>60% of the background	2–3 pixels	$1/2\;L_{B}\;>\;L_{D}$ > 2/3

**Table 2:** CP80-4-M-500 specification.

Parameter	Information
Resolution	2304 × 1720
Max Framerate (8 bit)	500
Active sensor area	16.13 mm × 12.04 mm
Pixel size in μm	7 μm × 7 μm
Shortest exposure time	2 ms
Sensitivity	9 V/lux.s
Storage format	BMP, JPG, TIFF, AVI

**Table 3:** Evaluation index of displacement extraction for the checkerboard background.

Algorithm	RMSE	Consumed Time	Accuracy
L–K optical flow	4.0434 pixels	0.823 s	98.60%
Farneback optical flow	0.6509 pixels	2.484 s	87.20%
Cross-correlation	0.0828 pixels	0.624 s	100%

**Table 4:** Evaluation index of displacement extraction for the artificial speckle background.

Algorithm	RMSE	Consumed Time	Accuracy
L–K optical flow	0.2511 pixels	1.261 s	98.37%
Farneback optical flow	0.1884 pixels	4.848 s	90.68%
Cross-correlation	0.1432 pixels	1.024 s	100%

## Data Availability

Not applicable.
